# Large Mechanosensitive Thermoelectric Enhancement in Metallo-Organic Magnetic Molecules

**DOI:** 10.1021/acs.nanolett.3c02569

**Published:** 2023-11-21

**Authors:** Munirah Alsaqer, Abdalghani H.S. Daaoub, Sara Sangtarash, Hatef Sadeghi

**Affiliations:** Device Modelling Group, School of Engineering, University of Warwick, CV4 7AL Coventry, United Kingdom

**Keywords:** molecular electronics, metallo-organic radicals, spin transport, phonon transport, molecular thermoelectricity, mechanosensitivity

## Abstract

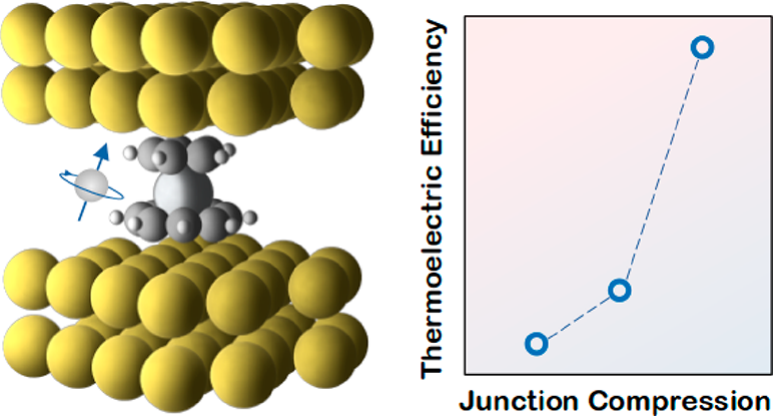

Organic materials are promising candidates for thermoelectric cooling and energy harvesting at room temperature. However, their electrical conductance (*G*) and Seebeck coefficient (*S*) need to be improved to make them technologically competitive. Therefore, radically new strategies need to be developed to tune their thermoelectric properties. Here, we demonstrate that *G* and *S* can be tuned mechanically in paramagnetic metallocenes, and their thermoelectric properties can be significantly enhanced by the application of mechanical forces. With a 2% junction compression, the full thermoelectric figure of merit is enhanced by more than 200 times. We demonstrate that this is because spin transport resonances in paramagnetic metallocenes are strongly sensitive to the interaction between organic ligands and the metal center, which is not the case in their diamagnetic analogue. These results open a new avenue for the development of organic thermoelectric materials for cooling future quantum computers and generating electricity from low-grade energy sources.

Molecular electronics provides an ideal platform for investigating the transport of charge and energy at the atomic and molecular scale.^1−3^ Due to the well-defined chemical structure of the molecules and their small size, the properties of molecules are dominated by quantum effects such quantum interference.^4−6^ In particular, studying thermoelectric properties of molecular junctions provide routes to investigate both charge and energy transport and to engineer their quantum properties to design novel materials with enhanced thermoelectric properties.^7−10^

Despite several decades of development, current thermoelectric materials that are mainly inorganic are not sufficiently efficient to convert waste heat to electricity at room temperature.^9^ Inorganic materials are not safe for the environment, as they are heavy, brittle, contain toxic elements, such as Pb, Bi, and Te, and their global supply is limited.^11^ A promising alternative is to use organo-metallic molecular scale ultrathin film materials to recover waste heat and economically generate electricity. This is because they involve a combination of heavy and light elements that can be used to filter heat. Also, their magnetic properties can be manipulated by the choice of organic ligands and metal centers.

To achieve high thermoelectric efficiency, materials with a high thermoelectric figure of merit, *ZT* = *GS*^2^*T*/κ are needed, where *S*, *G*, and κ are the Seebeck coefficient, the electrical conductance, and the thermal conductance, respectively. A high *ZT* is associated with low-κ, high-*G*, and high-*S*. The state-of-the-art world record *ZT* is slightly above 2 at high temperatures (e.g., 900 K) and about unity at room temperature in inorganic materials,^11,12^ which are toxic and suffer from a limited economically viable global supply.^13−15^ Materials with a high *ZT* are not easily obtainable, as *ZT* is constrained by the interdependency of *G*, *S*, and *κ*.^16^ However, molecular junctions are advantageous for thermoelectricity because they show low thermal conductance (less than 50 pW/K),^17^ and therefore, the main task of enhancing *ZT* in these materials is to increase their power factor *P* = *GS*.^2^

The organometallic complexes consist of transition metal atoms with 3*d* orbitals sandwiched between π-conjugated ligands show magnetic properties.^18,19^ These molecules have the advantage of being small with potentially high electrical conductance and thermal stability, and can form molecular films.^19−23^ Their extraordinary spin transport properties provide promising opportunities for the next generation quantum computing and memory storage.^24^ Such properties can also be useful for efficient conversion of heat to electricity; however, the organometallic molecules studied so far do not show a high power factor.^25−28^ This is mainly because these organometallic molecular junctions are closed-shell structures. Among organometallic complexes, metallocene has the advantage of being small and thus can lead to high conductance, but also can retain fantastic magnetic properties that can be used for thermoelectric enhancement.^29−31^ However, this has been unexplored.

In this paper, we show the enhancement of a thermoelectric figure of merit in radical organometallic complexes and demonstrate that their *ZT* is mechanosensitive and can be enhanced significantly by couple of orders of magnitude when the junction is compressed slightly. We investigated the thermoelectric and mechanosensitive properties of two organometallic complex (Cp) molecules, namely, paramagnetic CpTi(cot) (**1** in Figure 1) and diamagnetic CpTi(cht) (**2** in Figure 1). Both of these molecules has been shown to be stable^19^ and includes a titanium atom sandwiched between a η^5^-cyclopentadienyl and a η^7^-cycloheptatraene in CpTi(cht) and a η^5^-cyclopentadienyl and a η^8^-cyclooctatetraene in CpTi(cot), as shown in Figure 1. Consequently, CpTi(cht) is a closed shell molecule, while the spin multiplicity in CpTi(cot) is 2 (total spin 1/2).

**Figure 1:**
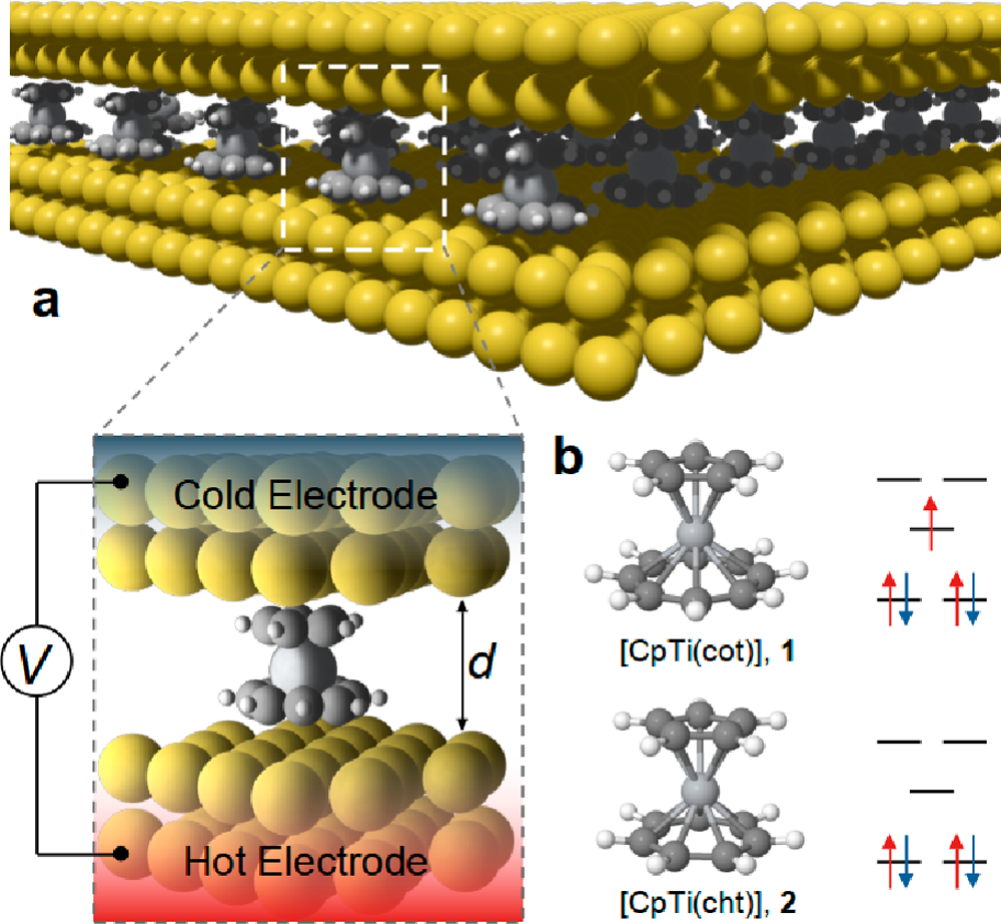
The molecular structure of metallocene junctions. (a) Schematic diagram of organometallic molecular structures connected to two hot and cold gold electrodes. (b) Molecular structures and energy level diagram of CpTi(cot) and CpTi(cht).

To study the quantum spin transport of junctions formed by molecules **1** and **2**, we first found the ground-state geometries of each junction using the SIESTA^32^ implementation of Density Functional Theory (DFT). We then obtained the spin-polarized mean-field Hamiltonian of each junction from DFT and combined it with our transport code GOLLUM^33,34^ to calculate the transmission coefficient *T*^σ^(*E*) for the majority (spin-up, σ = ↑) and minority (spin-down, σ = ↓) spins traversing from one gold electrode to the other (see Computational Methods for details).

Figure 2a(left) shows spin-dependent *T*^σ^(*E*) of junctions formed by paramagnetic molecule **1**. The transport is dominated by majority spins (green line) and the resonances due to the singly occupied molecular orbital (SOMO) and singly unoccupied molecular orbital (SUMO) are both due to spin-up electrons. The orbital calculations show that these resonances are due to the *d*-orbital of Ti (inset of Figure 2a(left)). This is in contrast with most organic radicals,^29,31,35−37^ where the radical feature is due to the polarization of *p*-orbitals and consequently the SOMO and SUMO resonances are due to spin-up and spin-down electrons, respectively.

**Figure 2:**
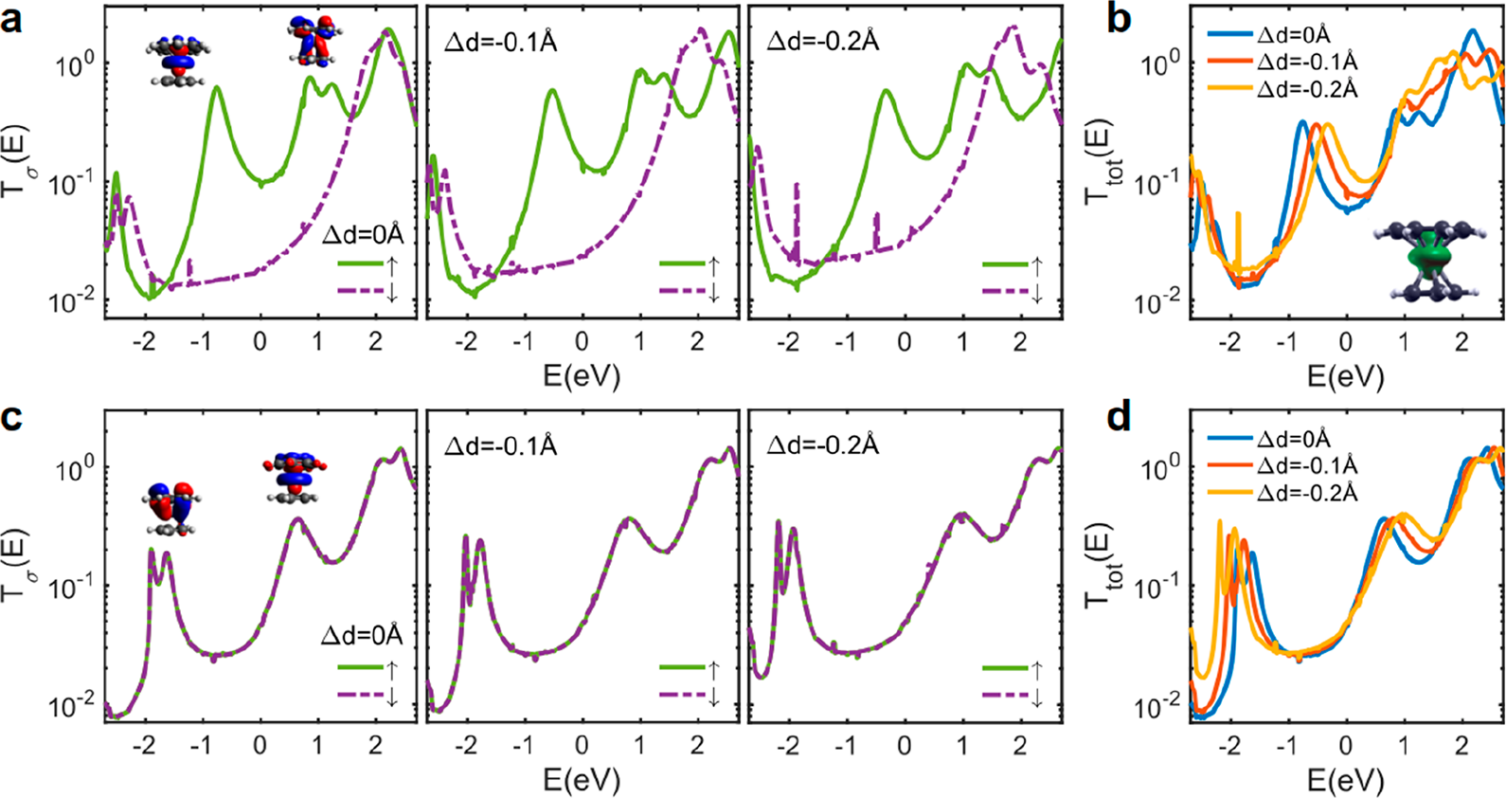
Spin transport through CpTi(cot) and CpTi(cht). Spin-dependent DFT transmission coefficient *T*(*E*) for spin-up and spin-down electrons for (a) CpTi(cot) and (c) CpTi(cht) as a function of compression Δ*d*. Total transmission for (b) CpTi(cot) and (d) CpTi(cht) for a junction with different compressions Δ*d* = 0, −0.1, and −0.2 Å. *E* = 0 eV denotes the DFT Fermi energy. Inset (a, c) shows spin orbitals for the frontier resonances. Inset (b) shows spin density in CpTi(cot).

Next, we studied how the spin transport through paramagnetic molecule **1** is affected by compressing it. For this, we started from the ground state geometry of the molecule in the junction and decreased the distance between the two electrodes by a small amount Δ*d* = −0.1 Å (1% of the junction length). We then fixed the position of the gold electrodes and let the structure relax to its ground state. As a result, the distance between the organic ligands and the Ti atom decreases. For the new junction configuration, we then calculated *T*^σ^(*E*). Figure 1a(middle and right) shows *T*^σ^(*E*) when Δ*d* = −0.1 and −0.2 Å. We found that such small changes in *d* lead to large variations in the position of the SOMO resonance (c.a. 0.5 eV), while the changes in the position of SUMO are much smaller (Figure 2a). The SOMO orbital is mainly affected because the spin density calculation of paramagnetic **1** shows that spin density is mainly localized on the Ti atom, and its distribution is very similar to the SOMO state (inset of Figure 2b).

Figure 2b shows the total transmission (*T*_tot_*= T*_*↑*_/2 + *T*_*↓*_/2) of paramagnetic molecule **1**. As a result of large changes in the position of SOMO, the amplitude of *T*_tot_ increases around the DFT Fermi energy, which is expected to enhance the electrical conductance because conductance is proportional to the total transmission^34^ (*G* ∝ *G*_0_*T*(*E*_F_), where *E*_F_ is the Fermi energy of the electrodes and *G*_0_ is the conductance quantum). To investigate if these properties are unique to open-shell molecule **1**, we also studied the effect of changes of *d* on the transport properties of closed-shell **2**, as shown in Figure 2c,d. First, we note that the spin transmissions are identical for closed-shell molecule **2** (Figure 2c), as expected, because the Hamiltonian for spin-up and spin-down are not spin polarized. Second, the changes in *d* have a smaller effect on the position of HOMO and LUMO resonances (Figure 2d), and both HOMO and LUMO resonances move away from the DFT Fermi energy (*E* = 0 eV in Figure 2c). As a result, *T*_tot_ is not affected significantly in **2**.

Next, we studied the effect of changes of *d* on the thermoelectric performance of molecules **1** and **2**. For this, from the calculated total transmission functions (Figure 2b,d), we calculated electrical conductance and Seebeck coefficient of **1** and **2**. Figures 3a,b show the room temperature Seebeck coefficients of **1** and **2**, respectively, as a function of Δ*d* and the position of the Fermi energy of the electrodes, *E*_F_. For a wide range of Fermi energies around the DFT Fermi energy (*E*_F_ = 0 eV), *S* increases in radical **1**, while it decreases in closed-shell **2**. The conductance also increases in **1**, while it is almost constant in **2** (see Figure 3c and Figure S2 of the Supporting Information). As a result, room temperature power factor (*P* = *GS*^2^) is expected to enhance significantly with *d* in **1** for a wide energy range around the DFT *E*_F_ (Figure 3c).

**Figure 3:**
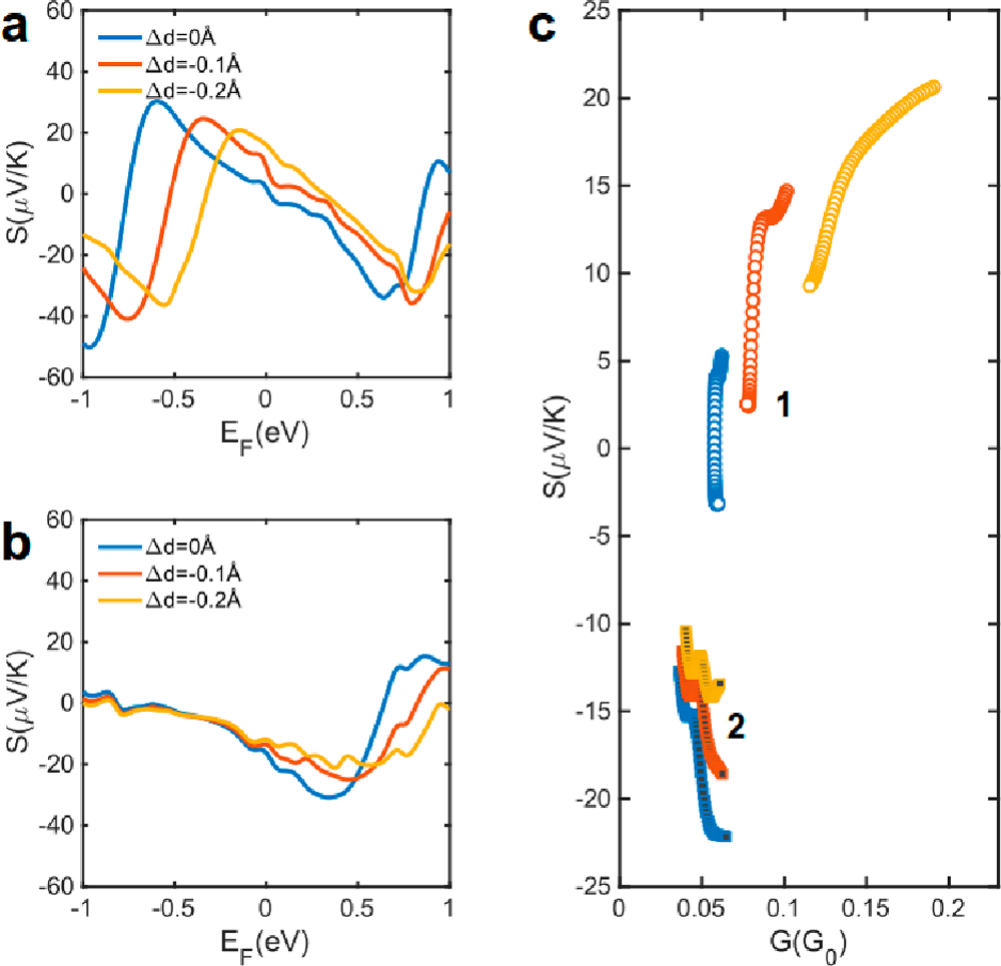
Thermoelectric coefficient of CpTi(cot) and CpTi(cht). Room-temperature Seebeck coefficient *S* for (a) CpTi(cot) and (b) CpTi(cht) as a function of electrodes Fermi energy *E*_F_. (c) *S* as a function of *G* for Δ*d* and different Fermi energies around *E*_F_ = 0 eV.

To understand the mechanosensitivity of thermoelectric coefficients in open-shell molecule **1**, we analyzed Mulliken charge density for each spin in molecules **1** and **2** as a function of Δ*d*, as shown in Table 1. The charge transfer between Ti and organic ligands are not the same for spin-up and spin-down electrons in paramagnetic molecule **1** in contrast to diamagnetic molecule **2**. The spin-up density of electrons on Ti decreases in **1**, while the spin-down density increases when ligands get closer to Ti. This is in contrast to **2**, where both spin-up and spin-down densities increase with Δ*d*. As a result of these charge transfers, the SOMO resonance in **1** moves toward *E*_F_ (Figure 2b), while HOMO and LUMO of **2** and SUMO of **1** move away from *E*_F_ (Figure 2d). This decreases the SOMO–SUMO transport gap of **1**, while the HOMO–LUMO gap in **2** increases with Δ*d*. We found that the SOMO resonance in **1** rapidly moves toward the Fermi energy, because this resonance is due to the localized states on the Ti metal center (Figure S5b), which experiences a reduction in their spin-up density upon compression. The changes in the position of the frontier spin orbitals leads to an enhancement of the power factor in **1**. This is because the electrical conductance is proportional to the amplitude of the transmission coefficient, while the Seebeck coefficient is proportional to its slope.^34^ This means that if a transport resonance moves closer to the Fermi energy of electrodes, a simultaneous enhancement of *S* and *G* and consequently a significant enhancement of the power factor is expected.

**Table 1:** Mulliken Charge on the Ti Atom in **1** and **2**

	**1**		**2**
Δ*d* (Å)	σ = ↑	σ = ↓	σ = ↑,↓
0	2.71	1.36	2.02
–0.1	2.68	1.48	2.05
–0.2	2.59	1.57	2.06

Next, we calculated the thermal conductance and full *ZT* for **1** and **2**. Heat is transmitted by both electrons and phonons.^38^Figure S6c,d shows the thermal conductance due to electrons (κ_e_) obtained from *T*_tot_(*E*) in Figure 2b,d. To calculate the thermal conductance due to phonons (κ_p_), we used material-specific *ab initio* calculations. As discussed in Computational Methods, we calculated the transmission coefficient of phonons *T*_p_(ω) with the energy ℏω traversing through **1** and **2** from one electrode to the other (Figure 4a,b) and used it to calculate κ_p_. Figure 4c,d shows κ_p_ for junctions formed by **1** and **2**, respectively. As the junctions are compressed, κ_p_ reduces for both **1** and **2** because the phonon transport resonances move down in energy and are suppressed by compression (Figure 4a,b). This reduction of κ_p_ is expected to further enhance the thermoelectric efficiency by compression. We also found that the amplitudes of κ_p_ and κ_e_ around the DFT Fermi energy are comparable. This is in contrast to other molecular junctions reported so far, where κ_p_ is usually higher than κ_e_.^17^ This suggests that in molecular junctions formed from metallocenes, the main task to enhance thermoelectric efficiency lies in improving *S* and *G*.

**Figure 4:**
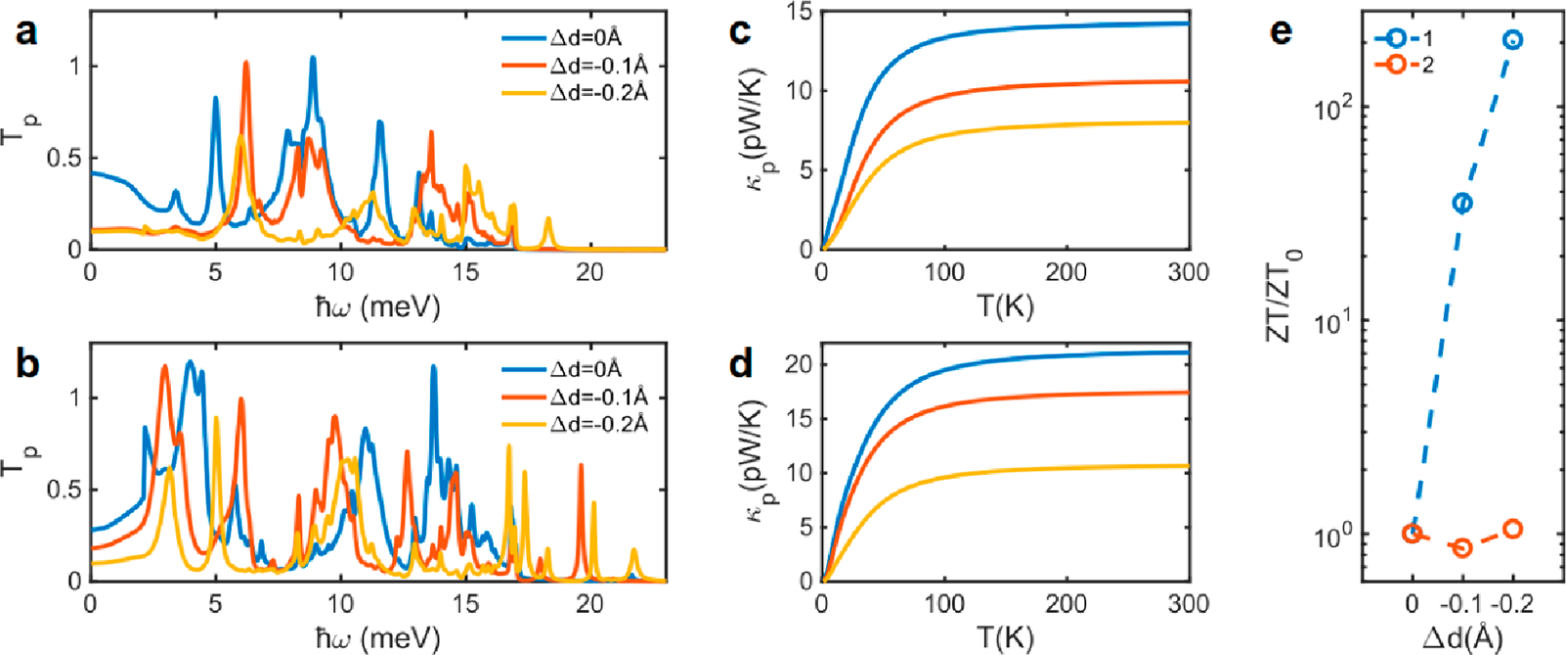
Thermal conductance due to phonons and thermoelectric figure of merit *ZT* of CpTi(cot) and CpTi(cht). Phonon transmission coefficient for (a) CpTi(cot) and (b) CpTi(cht) as a function of phonons with energy ℏω. Phonon thermal conductance for (c) CpTi(cot) and (d) CpTi(cht) as a function of temperature. (e) *ZT* of **1** and **2** as a function of the compression Δ*d* at DFT Fermi energy *E*_F_ = 0 eV relative to *ZT* of noncompressed molecule (*ZT*_0_).

Simultaneous enhancement of *G* and *S* and suppression of κ in paramagnetic molecule **1** leads to a significant enhancement of the thermoelectric figure of merit (*ZT*) as a function of compression for a wide energy range around the DFT Fermi energy (Figure S6). For example, upon compression, Δ*d*, the room temperature *ZT* at the DFT Fermi energy enhances by more than 2 orders of magnitude in **1**, while it remains almost constant in **2** (Figures 4e and S6d of the Supporting Information). These *ZT* values are higher than those recently measured for organic diamagnetic single molecules.^39^

To demonstrate that the enhancement of thermoelectric coefficients is a general feature of paramagnetic metallocenes, we also studied *S* and *G* in paramagnetic vanadocene (V(C_5_H_5_)_2_), as shown in Figure S7 of the Supporting Information. The SOMO and SUMO resonances in vanadocene are due to the spin-up and spin-down electrons, respectively (Figure S7c). Unlike CpTi(cot), SUMO is closer to the DFT Fermi energy and moves toward the Fermi energy rapidly (ca. 1 eV) upon less than 2% compression of the junction (Figure S7f), leading to a simultaneous increase in *G* and *S* (Figure S7g). Because SUMO is closer to the Fermi energy, the sign of *S* is negative. This is useful because tandem thermoelectric devices (Figure S8) require materials with both negative and positive *S* (*n*-type and *p*-type). These *n*-type and *p*-type paramagnetic metallocenes have very similar lengths, providing an opportunity to form a more uniform tandem device.

It is worth noting that using compression to shift the position of resonances in paramagnetic molecules is advantageous compared to other methods, such as electrostatic gating or electrochemical gating. This is because in diamagnetic molecular junctions, where the HOMO and LUMO are far from the Fermi energy, a large gate voltage must be applied to move the resonances close to the Fermi energy, which is practically challenging.^40^ Electrochemical gating is also undesirable for a thermoelectric device because the analyte used in these devices will form a parallel path for heat conduction, leading to poor thermoelectric performance.

In summary, we studied the thermoelectric properties of paramagnetic and diamagnetic metallo-organic molecules and their mechanosensitivity. We demonstrated that the transport resonances are more sensitive to the junction compression in paramagnetic **1** compared to diamagnetic **2**. We showed a significant enhancement of power factor and *ZT* by more than 2 orders of magnitude in **1** compared to **2** as a result of a small compression of 2% of the junction length. These results provide further insight into the charge transport mechanism and its sensitivity to external mechanical stimuli in paramagnetic organometallic molecules. They also promise new avenues to enhance the thermoelectric properties of these materials by tuning their packing between two electrodes. Furthermore, the sensitivity of electronic properties of paramagnetic metallocenes to compression makes them attractive for other applications, such as electromechanical molecular switching and ultrasensitive pressure sensing.

## Computational Methods

### Density Functional Theory Calculations

The geometry of each structure studied in this paper was relaxed to the force tolerance of 10 meV/Å using the *SIESTA*(32) implementation of DFT, with a double-ζ polarized basis set (DZP) and the Generalized Gradient Approximation (GGA) functional with Perdew–Burke–Ernzerhof (PBE) parametrization. A real-space grid was defined with an equivalent energy cutoff of 250 Ry. We then calculate spin-polarized molecular orbitals and the spin density of the gas-phase molecules.

### Spin Transport

To calculate the electronic properties of the device from the converged DFT calculation, the underlying spin polarized mean-field Hamiltonian *H*^σ^ was obtained, where σ = *↑*, *↓*, and *↑*(*↓*) denote majority (minority) spin. *H*^σ^ was combined with our quantum transport code, *GOLLUM*.^33^ This yields the spin-dependent transmission coefficient *T*^σ^(*E*) for electrons of energy *E* (passing from the source to the drain) via the relation *T*^σ^(*E*) = Tr(Γ_*L*_^σ^(*E*)*G*_σ_^*R*^(*E*)Γ_*R*_^σ^(*E*)*G*_σ_^*R*†^(*E*)), where Γ_*L*,*R*_^σ^(*E*) = *i*(∑_*L*,*R*_^σ^(*E*) – ∑_*L*,*R*_^σ†^(*E*)) describes the level broadening due to the coupling between left *L* and right *R* electrodes and the central scattering region, Σ_*L*,*R*_^σ^(*E*) is the retarded self-energy associated with this coupling, and *G*_σ_^*R*^ = (*ES* – *H* – ∑_*L*_^σ^ – ∑_*R*_^σ^)^−1^ is the retarded Green’s function, where *H*^σ^ is the Hamiltonian and *S* is the overlap matrix obtained from *SIESTA* implementation of DFT. The total transmission is then calculated from *T*(*E*) = (*T*^*↑*^ + *T*^*↓*^)/2.

### Phonon Transport

Following the method described in refs (34 and 41), a set of *xyz* coordinates were generated by displacing each atom from the relaxed *xyz* geometry in the positive and negative *x*, *y*, and *z* directions, with δ*q*′ = 0.01 Å. The forces *F*_*i*_^*q*^ = (*F*_*i*_^*x*^, *F*_*i*_^*y*^, *F*_*i*_^*z*^) in three directions *q*_*i*_ = (*x*_*i*_, *y*_*i*_, *z*_*i*_) on each atom were then calculated and used to construct the dynamical matrix *D*_*ij*_ = *K*_*ij*_^*qq*′^/*M*_*ij*_, where the mass matrix  and *K*_*ij*_^*qq*′^ = [*F*_*i*_^*q*^(δ*q*_*j*_^′^) – *F*_*j*_^*q*^(− δ*q*_*j*_^′^)]/2δ*q*_*j*_^′^ for *i* ≠ *j* were obtained from finite differences. To satisfy momentum conservation, the *K* for *i* = *j* (diagonal terms) is calculated from . The phonon transmission *T*_p_(ω) then can be calculated from the relation *T*_p_(ω) = *Trace*(Γ_*L*_^p^(ω)*G*_p_^*R*^(ω)Γ_*R*_^p^(ω)*G*_p_^*R*†^(ω)), where Γ_*L*,*R*_^p^(ω) = *i*(∑_*L*,*R*_^p^(ω) – ∑_*L*,*R*_^p†^(ω)) describes the level broadening due to the coupling to the left *L* and right *R* electrodes, ∑_*L*,*R*_^p^(ω) is the retarded self-frequency associated with this coupling, and *G*_p_^*R*^ = (ω^2^*I* – *D* – ∑_*L*_^p^ – ∑_*R*_^p^)^−1^ is the retarded Green’s function, where *D* and *I* are the dynamical and the unit matrices, respectively. The phonon thermal conductance κ_p_ at temperature *T* is then calculated from κ_p_(*T*) = (2π)^−1^∫_0_^∞^ℏω*T*_p_(ω)(∂*f*_BE_(ω, *T*)/∂*T*)dω, where *f*_BE_(ω, *T*) = (*e*^ℏω/*k*_B_*T*^ – 1)^−1^ is the Bose–Einstein distribution function in which ℏ is the reduced Planck’s constant and *k*_B_ is the Boltzmann’s constant.

### Thermoelectric Properties

Using the approach explained in refs (34 and 41), the electrical conductance, *G* = *G*_0_*L*_0_, the electron contribution of the thermal conductance, κ_el_ = (*L*_0_*L*_2_ – *L*_1_^2^)/*hTL*_0_, and the Seebeck coefficient, *S* = −*L*_1_/*eTL*_0_, are calculated from the electron transmission coefficient *T*_*e*_(*E*), where the momentums *L*_*n*_ = ∫_–∞_^+∞^ d*E* (*E* – *E*_F_)^*n*^*T*_*e*_(*E*)(− ∂*f*_FD_(*E*, *T*, *E*_F_)/∂*E*), where *f*_FD_ is the Fermi–Dirac probability distribution function, *f*_FD_ = (*e*^(*E*–*E*_F_)/*k*_B_*T*^ + 1)^−1^, *T* is the temperature, *E*_F_ is the Fermi energy, *G*_0_ = 2*e*^2^/*h* is the conductance quantum, *e* is electron charge, and *h* is the Planck’s constant. The full thermoelectric figure of merit *ZT* is then calculated as *ZT*(*E*_F_, *T*) = *G*(*E*_F_, *T*)*S*(*E*_F_, *T*)^2^*T*/κ(*E*_F_, *T*), where κ(*E*_F_, *T*) = κ_e_(*E*_F_, *T*) + κ_p_(*T*) is the thermal conductance due to the electrons and phonons.

## Data Availability

The input files to reproduce simulation data can be accessed by contacting the authors.

## Supporting Information Available

Details of the calculations, molecular orbitals for all molecules, spin densities, additional transmission coefficient calculations, and thermoelectric properties of vanadocene (PDF)
