# Supplementary material for: Large Mechanosensitive Thermoelectric Enhancement in Metallo-Organic Magnetic Molecules
Source: Nano Lett. 2023 Nov 21;23(23):10719–24. doi: 10.1021/acs.nanolett.3c02569 (PMC10722535; doi:10.1021/acs.nanolett.3c02569)
Supplement: Supplementary file 1 — nl3c02569_si_001.pdf [file nl3c02569_si_001.pdf]

Supporting Information

Large Mechanosensitive Thermoelectric Enhancement in Metallo-Organic Magnetic Molecules

Munirah Alsaqer, Abdalghani H.S. Daaoub, Sara Sangtarash*, and Hatef Sadeghi*

Device Modelling Group, School of Engineering, University of Warwick, CV4 7AL Coventry, UK
* Sara.Sangtarash@warwick.ac.uk; Hatef.Sadeghi@warwick.ac.uk

Table S1. Frontier orbital table for the gas-phase structures

structure	HOMO-2	HOMO-1	HOMO	Gap	LUMO	LUMO+1	LUMO+2
Δd=0Å	-6.04 (ev)	-4.37(ev)	-4.37(ev)	2.42	-1.95(ev)	-0.28(ev)	-0.27(ev)
Δd=-0.1Å	-5.88 (ev)	-4.54 (ev)	-4.31 (ev)	2.51	-1.80(ev)	-0.23(ev)	-0.07(ev)
Δd=-0.2Å	-5.75(ev)	-4.65(ev)	-4.41(ev)	2.79	-1.62(ev)	-0.07(ev)	0.09(ev)

structure	HOMO-2	HOMO-1	HOMO	Gap	LUMO	LUMO+1	LUMO+2
Δd=0Å Spin up	-5.21 (ev)	-5.21(ev)	-3.19(ev)	1.99	-1.20(ev)	-1.20(ev)	-0.23(ev)
Δd=0Å Δd=0Å Spin down	-5.75 (ev)	-4.90 (ev)	-4.90 (ev)	4.53	-0.37(ev)	-0.27(ev)	-0.26(ev)
Δd=-0.1Å Spin up	-5.27(ev)	-5.02(ev)	-2.94(ev)	1.69	-1.25(ev)	-1.01(ev)	-0.06(ev)
Δd=-0.1Å Spin down	-5.65(ev)	-4.98(ev)	-4.77(ev)	4.31	-0.46(ev)	-0.33(ev)	-0.29(ev)
Δd=-0.2Å Spin up	-5.33(ev)	-5.08(ev)	-2.63(ev)	1.55	-1.08(ev)	-0.83(ev)	0.08(ev)
Δd=-0.2Å Spin down	-5.09(ev)	-4.85(ev)	-4.78ev)	4.43	-0.35(ev)	-0.28(ev)	0.17(ev)

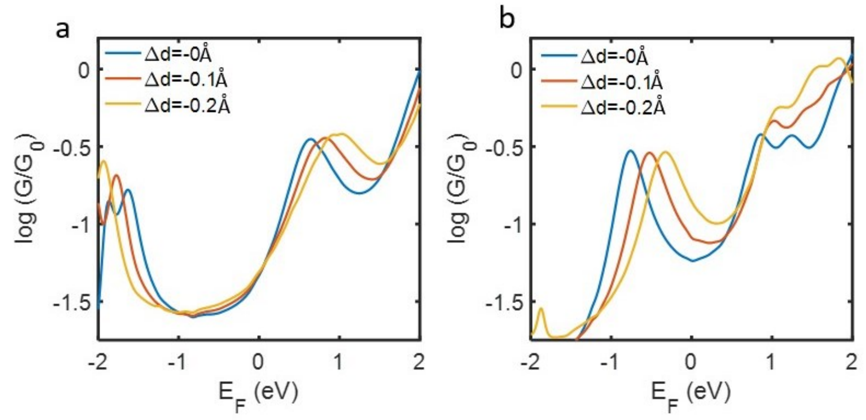

Figure S2. Electrical conductance at room temperature for (a), CpTi(cht) and (b) CpTi(cot). The bule curves correspond to the ground state structures while the red and orange curves correspond to 0.1Å and 0.2Å compression of the junctions, respectively.

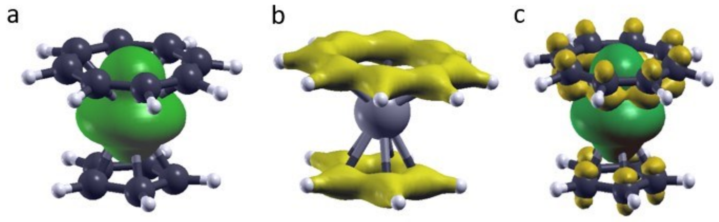

Figure S3. The real space distribution of spin density on CpTi(cot). (a) spin-up density, (b) spin-down density, (c) spin density for the ground-state CpTi(cot).

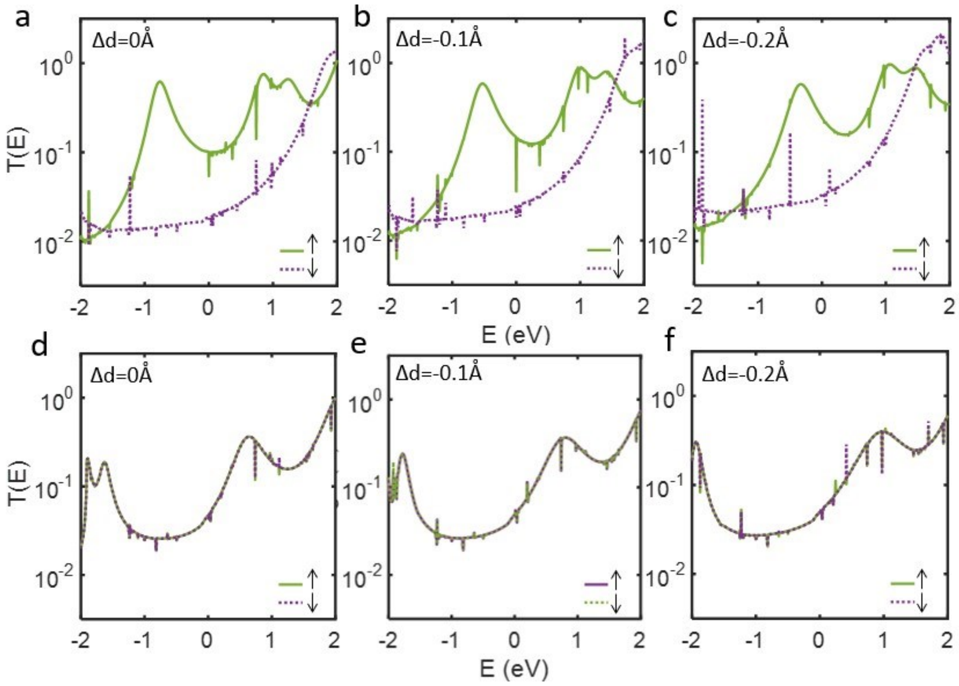

Figure S4. Spin transport through CpTi(cot) and CpTi(cht). The spin-dependent transmission coefficient $T(E)$ for spin-up and spin-down electrons for (a-c) CpTi(cot) and (d-f) CpTi(cht) as a function of the compression Δd. E=0eV denotes the DFT Fermi energy.

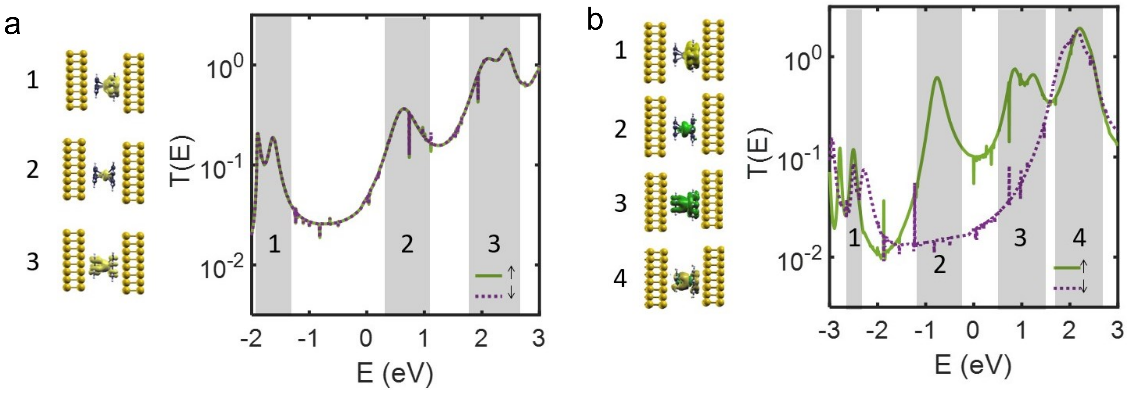

Figure S5. Local density of states for energy ranges shown by the grey colour for (a) CpTi(cht), and (b) CpTi(cot).

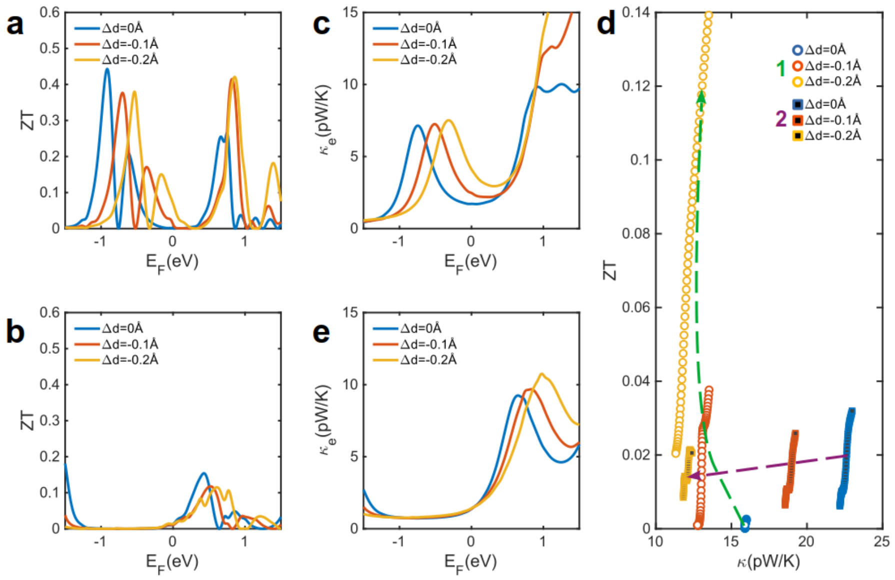

Figure S6. Thermoelectric figure of merit (ZT) of **1** and **2**. Room temperature ZT for (a) **1** and (b) **2** as a function of the electrodes' Fermi energy. Room temperature thermal conductance due to electrons (κ_e) for (c) **1** and (e) **2** as a function of the electrodes' Fermi energy. (d) Room temperature ZT of **1** and **2** as a function of the total thermal conductance for a range of Fermi energies around the DFT Fermi energy (E_F=0eV)

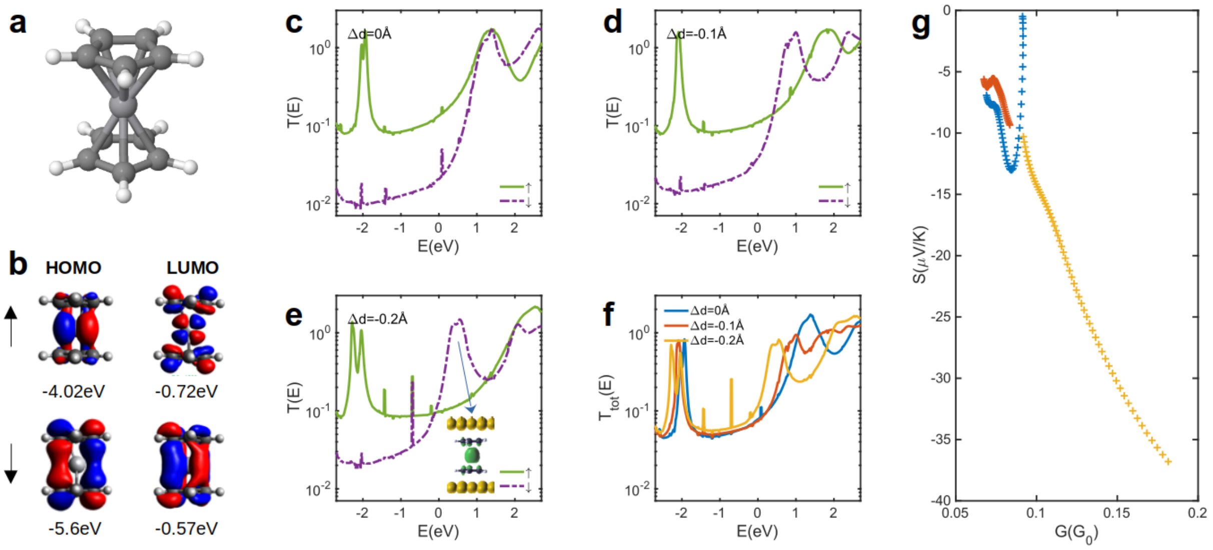

Figure S7. DFT transport and thermoelectric properties of $V(C_5H_5)_2$. (a) Molecular structure of the gas phase $V(C_5H_5)_2$, (b) Frontier orbitals for spin-up and spin-down electrons, (c-e) Transmission coefficient for each spin as a function of the compression Δ*d,* (f) the total transmission coefficient through $V(C_5H_5)_2$ sandwiched between the electrodes (inset of e), (g) Seebeck coefficient as a function of the conductance at room temperature and for a range of Fermi energies around the DFT Fermi energy. Inset of e shows LDOS for the energies around the transport resonance shown by the blue arrow.

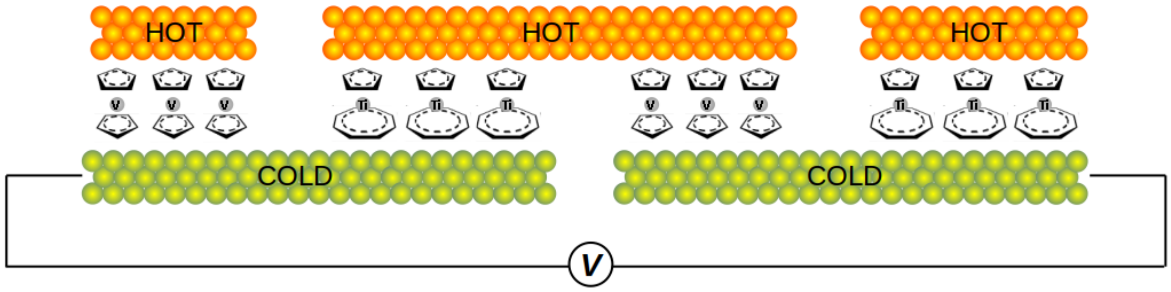

Figure S8. Schematic of a tandem thermoelectric device formed by paramagnetic metalocenes with the positive and negative Seebeck coefficients.

Calculation of forces between two parallel plates.

The force *F* between two parallel plates can be calculated from: $F = \varepsilon_0 V^2 \frac{A}{d^2}$ where $\varepsilon_0 = 8.85 \times 10^{-12}$ is the permittivity of free space, *V* is the voltage in volts (V), *d* is distance between the plates in meter (m) and *A* is the area of each plate in square meters (m²). The force between two parallel plates with a cross section of A=1nm^2 separated by d=1nm and under 1V is 0.009 nN. From our DFT calculations, the total energy of junctions formed by the molecule's changes by at least 0.13eV with 0.1Å displacement (Table S2). This means that F=d*E*/d*d* = 0.2nN is needed to make this displacement. This is 20 times more than the force applied to a molecule as a result of 1V bias voltage and 20,000 time more than that of generated by typical thermal voltage 1mV for ΔT=40K[1]. Therefore, the force generated as a result of thermal voltage will have a negligible effect on the result presented in this paper.

Table S2. The changes in the total energy of **1** and **2** compared to their ground state energy (E_{tot} at Δd = 0Å.)

Δd (Å)	ΔE_{tot} of **1** (eV)	ΔE_{tot} of **2** (eV)
0	0	0
-0.1	0.133	0.187
-0.2	0.496	0.706

References

1. Miao, R. *et al.* Influence of Quantum Interference on the Thermoelectric Properties of Molecular Junctions. *Nano Lett.* **18**, 5666–5672 (2018).
